# Genetic and molecular mechanisms of hydrocephalus

**DOI:** 10.3389/fnmol.2024.1512455

**Published:** 2025-01-07

**Authors:** Xuehai Deng, Yiqian Chen, Qiyue Duan, Jianlin Ding, Zhong Wang, Junchi Wang, Xinlong Chen, Liangxue Zhou, Long Zhao

**Affiliations:** ^1^Department of Neurosurgery, Affiliated Hospital of North Sichuan Medical College, Nanchong, China; ^2^School of Clinical Medicine, North Sichuan Medical College, Nanchong, China; ^3^School of Dentistry, North Sichuan Medical College, Nanchong, China; ^4^Department of Neurosurgery, the First Affiliated Hospital of Chongqing Medical University, Chongqing, China; ^5^Department of Neurosurgery, West China Hospital, Sichuan University, Chengdu, China

**Keywords:** hydrocephalus, genetic abnormality, animal model, molecular changes, cerebrospinal fluid

## Abstract

Hydrocephalus is a neurological condition caused by aberrant circulation and/or obstructed cerebrospinal fluid (CSF) flow after cerebral ventricle abnormal dilatation. In the past 50 years, the diagnosis and treatment of hydrocephalus have remained understudied and underreported, and little progress has been made with respect to prevention or treatment. Further research on the pathogenesis of hydrocephalus is essential for developing new diagnostic, preventive, and therapeutic strategies. Various genetic and molecular abnormalities contribute to the mechanisms of hydrocephalus, including gene deletions or mutations, the activation of cellular inflammatory signaling pathways, alterations in water channel proteins, and disruptions in iron metabolism. Several studies have demonstrated that modulating the expression of key proteins, including TGF-β, VEGF, Wnt, AQP, NF-κB, and NKCC, can significantly influence the onset and progression of hydrocephalus. This review summarizes and discusses key mechanisms that may be involved in the pathogenesis of hydrocephalus at both the genetic and molecular levels. While obstructive hydrocephalus can often be addressed by removing the obstruction, most cases require treatment strategies that involve merely slowing disease progression by correcting CSF circulation patterns. There have been few new research breakthroughs in the prevention and treatment of hydrocephalus.

## 1 Introduction

Hydrocephalus is a common neurological condition and is defined as the progressive distension of the brain ventricular system induced by a disorder of cerebrospinal fluid (CSF) microcirculatory homeostasis and characterized by an abnormal accumulation of CSF (Kahle et al., 2016; Hochstetler et al., 2022). Hydrocephalus is typically classified as either obstructive hydrocephalus or communicating hydrocephalus based on to the characteristics of CSF circulation. While obstructive hydrocephalus can often be resolved by removing the obstruction (Mekbib et al., 2023), other forms of hydrocephalus must be treated by slowing disease progression and correcting CSF circulation patterns. However, there have been few significant clinical breakthroughs in the treatment of the underlying causes of hydrocephalus, likely due to the condition’s insidious and complex etiology.

Hydrocephalus can also be categorized as congenital, acquired, or idiopathic normal pressure hydrocephalus based on its underlying cause (Rekate, 2009). Specifically, the pathogenesis of congenital hydrocephalus, which is often linked to aqueductal stenosis, involves various molecular changes that are associated with genes that regulate brain growth and development, with approximately 40% of congenital hydrocephalus cases having a genetic origin (Kundishora et al., 2021; Duy et al., 2022; Zhang et al., 2006). Most cases of secondary hydrocephalus may be attributed to a single primary cause, such as stroke, traumatic brain injury, brain tumor, infection, or craniectomy, involving multiple pathological processes, such as abnormal cerebrospinal fluid secretion and absorption, abnormal subarachnoid circulation, and decreased cerebral venous compliance (Xu, 2016; Chen et al., 2017). These pathological changes are also involved in signaling pathways such as inflammation, fibrosis, ion and transport channels, and vascular injury and reconstruction (Yamashiro, 2022; Huang et al., 2023; Toft-Bertelsen et al., 2022; Claassen and Park, 2022). Moreover，although a variety of hydrocephalus-related proteins have been found to be associated with idiopathic hydrocephalus, the etiology of idiopathic hydrocephalus has yet to be fully elucidated (Ishida et al., 2023).

In recent years, advances in research on the genetic and molecular mechanisms of hydrocephalus have led to the development of drugs and gene therapies targeting these mechanisms, which have shown promising effects in preclinical studies. These findings suggest that focusing on these genetic loci and molecular targets could be a potential approach for improving the clinical treatment of hydrocephalus (Davy and Robinson, 2003). In this review, we focus on the genetic and molecular changes involved in the pathogenesis of hydrocephalus in studies in animals and humans, and we discuss the potential value of these molecules in terms of diagnosing and treating hydrocephalus.

## 2 Genetic disorders of hydrocephalus

A substantial body of research has identified numerous genetic abnormalities associated with hydrocephalus, and the various genetic loci have been identified through studies conducted on animal models of hydrocephalus (Table 1). In recent years, with the development of genomics and molecular biology technologies, significant breakthroughs have been made in the study of genes associated with congenital hydrocephalus in humans (Table 2). Genetically abnormal hydrocephalus models exhibit histomorphologic alterations that closely resemble those observed in human congenital hydrocephalus, making these models valuable tools for investigating the genetic and pathological mechanisms underlying this condition. The majority of current research on genetic abnormalities associated with hydrocephalus has been conducted in rodent models, particularly rats and mice. The key genetic alterations identified in these models have been further validated in other animal systems. These animal models of hydrocephalus not only share significant histopathological features with human cases but also provide critical insights into the genetic and pathogenic processes contributing to brain injury. Compared with human models, animal models of hydrocephalus present many histopathological features, thus providing critical insights into the genetic and pathogenic processes contributing to brain injury (Zhang et al., 2006).

**Table 1:** Gene loci in animal models of hydrocephalus.

Disorder	Genetic locus	Genetic trait	Species/strain	Reference (s)
Congenital: defective neural cell adhesion/ stenosis of the aqueduct of Sylvius/agenesis of corpus callosum	L1CAM	X-linked /Xq28	Mouse/ Zebrafish	Adle-Biassette et al. (2013), Jouet et al. (1993), Okamoto et al. (2004), Willems et al. (1987), Bousquet et al. (2021), Wang et al. (2021), and Etchegaray et al. (2020)
Perturbation of growth factor signaling for cell function	TGFB, IGFBP-1, FGF-2, SOCS1	Chr19, Chr11, Chr4, Chr16	Rat	Zhang et al. (2006), Li et al. (2005), Doublier et al. (2000), Ohmiya et al. (2001), and Krebs et al. (2004)
Defective ependymal cell migration and proliferation /enhanced Notch signaling activity/aqueduct stenosis	Rnd3	Chr2	Mouse	Lin et al. (2013) and Jie et al. (2015)
Defective mesenchymal defective /cell PC and SCO /collapse of the cerebral aqueduct	Msx1, CYP2J2, RFX4_v3	Chr4, Chr1, Chr7	Mouse	Zhang et al. (2006) and Blackshear et al. (2003)
Defective differentiation of arachnoid cells/ obstruction of the interventricular aqueducts	Mf1, FREAC3		Mouse	Zhang et al. (2006) and Kume et al. (1998)
iNPH: dysfunction of the glymphatic pathway and sub-ischemia	AQP4, Dp71	Chr18	Mouse	Eide and Hansson (2018) and Zhao et al. (2022)
Congenital/obstructive: defective ependymal	Mdnah5, VANGL1, 2, KIF7, SMARCC1	Chr15, Chr1	Mouse	Wagner et al. (2003) and Banizs et al. (2005)
Defective embryo development and ventricular size	Vent8a, Vent4b, Vent7c	Chr4, Chr7, Chr8	Mouse	Zhang et al. (2006) and Zygourakis and Rosen (2003)
Defective cellular membrane fusion /abnormal development of the neuronal cells	a-SNAP, VAMP-7	Chr7, Chr2	Mouse	Hong et al. (2004) and Chae et al. (2004)
Defective brain development and edematous periventricular white matter	Otx2	Chr14	Mouse	Makiyama et al. (1997)
Hypersecretory: overproduction of CSF by choroid plexus	E2f5, Tg737orpk	Autosomal recessive	Mouse	Wagner et al. (2003), Banizs et al. (2005), Lindeman et al. (1998), and Putoux et al. (2011)
Cilia/flow/neural tube, and resultant closure of aqueduct	TRIM71	Chr9, Chr13	Mouse	Lindeman et al. (1998), Putoux et al. (2011), Furey et al. (2018), Jin et al. (2020), Kibar et al. (2011), Mastromoro et al. (2021), Kundishora et al. (2021), andLiu et al. (2023)
Defective cilia orientation/aberrant CSF flow	CCDC88C	Chr14	Zebrafish	Marguet et al. (2021) and Ohata et al. (2014)
SCO abnormalities/cerebral aqueduct closure/corpus callosum absence/free radical damage/abnormal cerebral hemisphere formation	Cck, Nfix, Xdh, Gsta1, Pax-6, Fkhr	Chr9,Chr8, Chr17,Chr9, Chr2	Rat	Zhang et al. (2006), Miller et al. (2006),Oi et al. (1996), Somera and Jones (2004), and Somera and Jones (2002)
Extracellular matrix disruption	TIMP-1, TGFB1	Chr7	Rat	Zechel et al. (2002)
Mutations in PI3K - Akt - mTOR signaling pathway genes	HERC1, FOXJ1, FMN2, SMARCC1, TRIM71, PTCH1	Chr9, Chr11, Chr1, Chr9, Chr9, Chr13	Mouse	Mashimo et al. (2009), Furey et al. (2018), Jacquet et al. (2009), Lian et al. (2019), and Gavino and Richard (2011)
Abnormal development of neural stem cells/abnormal role of ventricular membrane cell maintenance/Impaired differentiation and ciliation of ependymal cells	SOX9	Chr11	Mouse	Scott et al. (2010)
Interference with RhoA pathway /abnormal cortical development /abnormal neuronal migration	ADGRG1	Chr8	Mouse	Luo et al. (2011)
Diminished response to neurotrophic factors	KIDINS220	Chr2/Chr12	Mouse	Cesca et al. (2012)
Peripheral neuropathy/ agenesis of the corpus callosum / aqueductal stenosis	SLC12A6	Chr2	Mouse	Howard et al. (2002)
Uncoordinated movement and reduce the amplitude of cilia	Ccp5		Zebrafish	Lyons et al. (2013) and Pathak et al. (2014)
Block cilia movement and impair CSF fow	Efcab1	Chr8	Mouse	Sasaki et al. (2019)
Regulating the integrity structure and function of IDA and ODA	Pih1d3	X-linked	Rat	Zhang et al. (2023)
Destroy nervous system devel-opment by regulating calcium oncentration of synaptic	Calb2a, Calb2b	Chr7	Zebrafish	Bhoyar et al. (2019)
Perturb intracellular Mg2+ homeostasis	Slc41a1		Zebrafsih	Hurd et al. (2013)
Mediate neuronal apoptosis	Lgi1b	Chr12	Zebrafish	Teng et al. (2011)
Mediate ventricular epithelial cell apoptosis	Trx1	Chr4	Mouse	Yang et al. (2019)
Inhibit CNS injury	Ecrg4	Chr13	Zebrafish	Gonzalez et al. (2011)

L1CAM, L1 cell adhesion molecule; TGFB, transforming growth factor beta; IGFBP, insulin like growth factor blinding proteins; FGF, fibroblast growth factor gene; SOCS, suppressor of cytokine signaling; Rnd3, rho family GTPase 3, Msx1, muscle segment homeobox 1; CYP2j2, cytochrome P450 family2 subfamily j polypeptide2; RFX4-V3, regulatory factor X4 variant3; Mf1, homo sapiens flap structure-specific endonuclease 1; FREAC3, forlhead box C1; AQP4, aquaporin-4; Dp71, dystrophin-71; Mdnah5, dynein heavy chain 5 axonemal; Vangl, VANGL plannaar cell polarity; KIF7, kinesin family member 7; SMARCC1, SWI/SNF related matrix associated actin dependent regulator of chromatin subfamily c member1; a-SNAP, a-soluble NSF attachment protein; VAMP, vesicle-associated membrane protein; Otx2, orthodenticle homolog2; E2f5, e2f transcription factor 5; Tg737, intraflagellar transport 88 homolog; TRIM71, tripartite motifcontaining 71; CCDC88C, coiled-coil domain containing 88c; Cck, cholecystokinin; Nfix, nuclear factor l X type; Xdh, xanthine dehydrogenase; Gsta1, glutathione S-transferase alpha 1; Pax-6, paired box 6; Fkhr, forkhead box O1,TIMP-1, TIMP metallopeptidase inhibitor 1; HERC, endoplasmic reticulum-related comlex; FOXJ1, forkhead box J1; FMN2, formin 2; Ptch1, patched 1; SOX9, SRY (sex determining region Y)-box 9; Adgrg, adhesion G protein-coupled receptor; KIDINS220, kinase D-interacting substrate 220; SLC12A6, solute carrier family 12, member 6; Ccp5, cytosolic carboxypeptidase5; Efcab1, EF-hand calcium binding domain1; Pih1d3 Dnaaf6, dynein axonemal assembly factor 6; Calb calbindin; Slc41a1, solute carrier family 41 member 1; Lgi1b, leucine-rich, glioma inactivated 1b; Trx1, thioredoxin-1; Ecrg4, esophageal cancer related gene4; CSF, cerebrospinal fluid; CNS, central nervous system; IDA, inner dynein arms; ODA, outer dynein arms.

**Table 2:** Genetic loci in humans with hydrocephalus.

Disorder	Genetic locus	Genetic trait	Reference (s)
Congenital: defective neural cell adhesion/ stenosis of the aqueduct of Sylvius/agenesis of corpus callosum	L1CAM	X-linked /Xq28	Adle-Biassette et al. (2013),Jouet et al. (1993),Okamoto et al. (2004),Willems et al. (1987),Bousquet et al. (2021), Wang et al. (2021), Etchegaray et al. (2020), and Garcia-Bonilla et al. (2021)
Congenital/obstructive: defective ependymal/ cerebral ventriculomegaly/aqueductal stenosis/a variety of structural brain defects	SMARCC1	Chr3	Wagner et al. (2003), Banizs et al. (2005),Singh et al. (2023), andFurey et al. (2018)
Defective cilia orientation/aberrant CSF flow	CCDC88C	Chr14	(Marguet et al., 2021) and (Ohata et al., 2014)
Variable severity of hydrocephalus/ intellectual disability with prominent basal ganglia iron deposition/Aberrant vesicle trafficking	AP1S2	X-linked Xp22	Kousi and Katsanis (2016), Marguet et al. (2021), Yang et al. (2019), Saillour et al. (2007), and Marguet et al. (2021)
Disruption of the planar cell polarity pathway	MPDZ	Chr9	Al-Jezawi et al. (2018), Sotak and Gleeson (2012), and Marguet et al. (2021)
Hydrocephalus internus/chronic destructive airway disease/randomization of left/right body asymmetry	FOXJ1	Chr17q22–25	Wallmeier et al. (2019)

L1CAM, L1 cell adhesion molecule; THs, thyroid hormones; SMARCC1, SWI/SNF related matrix associated actin dependent regulator of chromatin subfamily c member1; CCDC88C, coiled-coil domain containing 88C; AP1S2, adaptor protein1 subunit sigma2; MPDZ, multiple PDZ domain protein; FOXC1, forkhead box C1.

### 2.1 Genetic loci in mice with hydrocephalus

In the mouse model, three quantitative trait loci (QTL) have been identified on chromosome (Chr) 8, Chr 4, and Chr 7; these loci were labeled Vent8a, Vent4b, and Vent7c, respectively (Zygourakis and Rosen, 2003). Vent8a, which is located on Chr 8 close to the markers D8Mit94 and D8Mit189, is the major QTL that controls variance in ventricular size. Vent4b, which is located on Chr 4 near D4Mit237 and D4Mit214, and Vent7c, which is located on Chr 7 between D7Mit178 and D7Mit191, affect ventricular size in the developing embryo.

Mouse models that have been reported to have congenital hydrocephalus include congenital hydrocephalus-1 (*hy1*), hydrocephalus-2 (*hy2*), hydrocephalus-3 (*hy3*), spontaneous congenital hydrocephalus (*ch*), hydrocephalus and hop gait (*Hyh*), hemorrhagic hydrocephalus (*Hhy*), and obstructive hydrocephalus (*oh*). *Hy1*, *hy2,* and *hy3* mice are autosomal recessive. *Hy1* and *hy2* mice are extinct, and no defective locus has been identified. These two strains share similar phenotypic characteristics, including dilation of the entire ventricular system within the first two weeks of life, which is potentially linked to conduit closure (Zhang et al., 2006; Kousi and Katsanis, 2016; Raimondi et al., 1976). Additionally, the *oh* strain, which carries an unidentified genetic locus, is autosomal recessive. In *oh* mice, the enlarged cerebral hemispheres compress the midbrain, leading to aqueductal occlusion and subsequent stenosis, ultimately culminating in obstructive hydrocephalus. Electron microscopy has revealed severe damage to subventricular cells and white matter, along with detachment of the ventricular meninges (Borit and Sidman, 1972). In contrast, a more extensive genetic analysis of *hy3* mice revealed a mutation in the Bdnf gene on chromosome 8 of the transgenic OVE459 mouse strain. The insertion of a transgene resulted in an exonic rearrangement of the Hydin gene in OVE459 mice. Furthermore, a single CG base pair deletion in exon 15 of the Hydin gene was discovered in *hy3* mice carrying a spontaneous *hy3* mutant allele. The Hydin protein, which is homologous to the actin-binding protein Caldesmon, is expressed specifically in the ciliated ependymal cell layer of the lateral, third, and fourth ventricles in *hy3* mice. It plays a crucial role in the formation, function, or maintenance of cilia, cilia-like structures, and ciliated epithelium (Davy and Robinson, 2003; Robinson et al., 2002; Lechtreck et al., 2008). Experimental evidence indicates an accumulation of lipids in the choroid plexus and ventricular membrane cells of *hy3* mice, implicating a potential role for Hydin in maintaining cellular homeostasis and/or signaling processes (Lawson and Raimondi, 1973). Hydin is also essential for ciliary motility, suggesting that impaired CSF flow due to reduced ciliary pulsation may serve as a primary initiating factor for hydrocephalus. Alternatively, ciliary dysfunction may contribute to alterations in the ventricular layer and subsequent changes in CSF production (Dawe et al., 2007).

Congenital hydrocephalus (*ch*) mice with autosomal recessive mutations have been shown to have a mutation in Foxc1 (Mf1) on mouse chromosome 13, which is a member of the conserved forkhead/winged helix transcription factor gene family expressed in embryonic tissues (Kume et al., 1998). Hydrocephalus in *ch* mice with Foxc1 mutations has been shown to be associated with multiple developmental defects, including abnormal differentiation of arachnoid cells in the meninges and the absence of certain skull bones (Hong et al., 1999). Interestingly, the deletion of several genes related to the structure and function of ventricular meningeal cilia, such as Mdnah5 (Ibanez-Tallon et al., 2002), Spa6 (Sapiro et al., 2002), and Rsph9 (Zou et al., 2020), which cause hydrocephalus in other mouse models, has not been implicated in congenital hydrocephalus.

A mouse model with autosomal recessive hydrocephalus and a characteristic hop gait (*Hyh*) exhibits ventricular enlargement and abnormal locomotion at birth. *Hyh* mice are characterized by marked ventricular dilatation, a small cerebral cortex, an interhemispheric cyst arising from the third ventricle, agenesis of the corpus callosum, and abnormal neural cell development; these mice eventually die due to worsening hydrocephalus (Batiz et al., 2006; Chae et al., 2004; Rodríguez-Pérez et al., 2024).This model is frequently utilized to investigate the pathogenesis of obstructive congenital hydrocephalus (Bronson and Lane, 1990). The Hyh gene, located on chromosome 7 near the Gpi-1 (glucose phosphate isomerase-1) locus, has been identified as Napa, a gene critical for the normal development of the neuroepithelium lining the ventricles. Mutations in Napa result in midbrain aqueduct obstruction by postnatal day 1, leading to severe hydrocephalus (Jiménez et al., 2001; Wagner et al., 2003). This obstruction triggers a cascade of neuropathological events, including myelin degeneration, glial activation, excitotoxic neurochemical environments, and edema (Páez et al., 2007; García-Bonilla et al., 2018); The Napa gene encodes the soluble N-ethylmaleimide-sensitive factor (NSF) attachment protein *α* (α-SNAP), which is essential for cell membrane fusion. Mutations in α-SNAP in *Hyh* mutants cause defects in vesicular transport, leading to pronounced abnormalities in F-actin organization, as well as in the distribution of α-connexin, β-connexin, and E-cadherin (Chae et al., 2004; Rodríguez-Pérez et al., 2024), and the disorder displays 100% penetrance, with the mutation present only in affected mice (Hong et al., 2004; Batiz et al., 2009; Chae et al., 2002).

Another mouse model of congenital hydrocephalus is the hemorrhagic hydrocephalus (*Hhy*) model. Homozygous *Hhy* mutants, which follow an autosomal recessive inheritance pattern, are characterized by intracranial hemorrhage, hydrocephalus, and subcortical heterotopia. Notably, these mice exhibit no histological abnormalities in the subarachnoid space or the choroid plexus. The *Hhy* gene locus has been mapped to mouse chromosome 12, and evidence suggests that *Ccdc85c*, located within a 1-Mb region between the *D12Mit28* and *D12Nds2* markers on chromosome 12, may be genetically disrupted in *Hhy* mutants (Mori et al., 2012; Express Group, 2010). Genetic deletion of Rho family guanosine triphosphatase 3 (Rnd3) and regulation of Notch signaling activity, resulting in the overgrowth of aqueduct ependymal cells, has been shown to be associated with aqueductal stenosis, which is a significant factor in congenital hydrocephalus (Lin et al., 2013). Therefore, inhibition of the Notch signaling pathway may be an effective target for treating hydrocephalus. Genetic studies on hydrocephalus in mice have provided a wealth of molecular insights into the pathogenesis of congenital hydrocephalus. These studies have significantly deepened our understanding of the related genetic factors, laying a solid foundation for uncovering the mechanisms behind hydrocephalus. Furthermore, findings from mouse models offer potential directions for the early diagnosis and personalized treatment of hydrocephalus in the future. In-depth translational research on these genes holds the promise of providing new clinical strategies, thereby advancing the precision diagnosis and treatment of congenital hydrocephalus.

### 2.2 Rats with hydrocephalus

Significant strains of congenital hydrocephalus include the Texas strain (HTX) and LEW/Jms in rats. Enlargement of the ventricular system occurs in HTX rats during late gestation, resulting from the closure of the cerebral aqueducts and a reduction in the secretory cells of the subcommissural organ (SCO). The SCO is a circumventricular organ located in the dorsal aspect of the cerebral aqueduct and is the source of sialylated glycoproteins that form Reissner’s fibers (RFs) and remain CSF-soluble (Ortloff et al., 2013). Moreover, SCO-spondin is a specific glycoprotein associated with neuronal maturation in the developing brain and has been shown to be correlated with both aqueduct stenosis and enlarged lateral ventricle size in HTX rats (Zhang et al., 2006; Chae et al., 2002).

QTL mapping of the progeny of a backcross of HTX rats with the nonhydrocephalic Fischer F344 strain revealed four loci for hydrocephalus on Chr 9 (peak markers D9Rat2), 10 (between markers D10Rat136 and D10Rat135), 11 (peak markers D11Arb2 and D11Rat46) and 17 (peak markers D17mit4 and D17Rat154) (Zhang et al., 2006; Jones et al., 2004). The Chr 9 locus closest to the TGIF (or the 5 V-TG-3 V interacting factor) encodes a gene that modulates the transforming growth factor-β (TGF-β) signaling pathway. TGF-β1 is a fiber factor that is associated with several fibrotic diseases and is significantly elevated in HTX rats (Li et al., 2005) In addition, the overexpression of TGF-β1 leads to fibrosis of the soft brain and arachnoid membranes as well as collagen deposition in the extracellular matrix (ECM) of the subarachnoid space (Yan et al., 2016; Yang et al., 2022). A further gene array study in the midbrain region of HTX rats with congenital hydrocephalus suggested that abnormal expression of cholecystokinin (Cck), nuclear factor 1/X (Nfix), three galactose-binding soluble lectins (Lgals3), glutathione s-transferase a type (Gsta1), Xdh (xanthine dehydrogenase), a tissue factor pathway inhibitor (Tfpi-2) and the fork-head transcription factor BF-1 (Fkhr) may be associated with hydrocephalus (Miller et al., 2006). Recently, Wagner et al. (2003) performed copy number analysis on H-Tx rats, revealing the pathophysiological mechanisms by which abnormal Ptpn20 gene expression is associated with the development of hydrocephalus in HTX rats. The expression of Ptpn20 mRNA was significantly lower in hydrocephalic HTX rats than in non-hydrocephalic HTX rats. In contrast, the expression of phosphorylated Na-K-Cl cotransporter 1 (pNKCC1) in the choroid plexus was significantly increased in mice with Ptpn20 gene deletion, suggesting that the overexpression of pNKCC1 on the epithelial cells of the cerebral choroid plexus, which results in excessive cerebrospinal fluid secretion, may be involved in hydrocephalus in HTX rats.

Folate is an essential nutrient for multiple metabolic pathways, and in the brain, only 5-methyl tetrahydrofolate (5mTHF) can freely cross the blood–brain barrier (Bailey and Gregory, 1999; Dunlevy et al., 2006; Pietrzik et al., 2010). It has been shown that disruptions in folate metabolism and methylation, particularly in male H-Tx rats, may contribute to the development and inheritance of hydrocephalus. Furthermore, bioactive folic acid has been demonstrated to significantly reduce the risk of hydrocephalus in these rats by modulating DNA methylation (Mori et al., 2012). HTX rats with hydrocephalus exhibit a decrease in hepatic and cerebral nuclear FDH and parallel increases in hepatic nuclear methylfolate and cerebral methylfolate at postnatal ages 5, 15, and 20. In parallel with the increase in folate-binding proteins and enzymes, 10-formyltetrahydrofolate dehydrogenase (FDH) fails to be secreted, resulting in the inability of cortical cells to access the available 5mTHF in cerebrospinal fluid supplemented with THF or 5fTHF (Jimenez et al., 2019). Another related study revealed that in *in vitro* culture, when cells were soaked with CSF containing high 5mTHF/FRa and low FDH levels, the growth of arachnoid tissue was overstimulated, leading to dysfunction of arachnoid tissue (Naz et al., 2016) and suggesting that CSF folate imbalance may also induce congenital hydrocephalus. LEW/Jms rats exhibit strains similar to those of HTX rats, and the inheritance of hydrocephalus in these rats may be autosomal recessive or semidominant. Nevertheless, none of the loci have been identified (Jones et al., 2003; Sasaki et al., 1983).

However, it is important to note that while these studies provide valuable insights, relevant genetic investigations have not yet been conducted to fully elucidate the underlying mechanisms. Future studies should focus on identifying specific genetic variants related to folate metabolism and their potential role in the inheritance of hydrocephalus, as well as exploring therapeutic strategies targeting folate signaling pathways.

### 2.3 Genetic alterations in hydrocephalus in other animals

Zebrafish genes share approximately 70% homology with human genes, making them highly amenable to genetic analysis and editing. Therefore, zebrafish are frequently used as model organisms to study the development of hereditary diseases affecting the ventricular system (Howe et al., 2013). Knockdown of the L1camb gene in zebrafish through the injection of control morpholinos or morpholinos targeting the splicing or translation of L1camb mRNA leads to axonal outgrowth defects and myelination abnormalities, ultimately resulting in hydrocephalus (Linneberg et al., 2019). Additionally, the camel gene has also been closely linked to hydrocephalus (Yang et al., 2021). Knocking out the wdr16 gene via antisense Morpholino injection induces hydrocephalus in zebrafish, although these animals still present with intact ciliary motility and no significant changes in the ventricular laminae, thus suggesting that the wdr16 gene plays a role in cilia-mediated cell polarization (Hirschner et al., 2007). The Atp1a3 gene, which is associated with Na+/K+ ATPase, is strongly correlated with hydrocephalus. Targeted knockdown of Atp1a3a or Atp1a3b results in abnormal dilation of the cerebral ventricles in zebrafish, likely due to ionic imbalance across the plasma membrane, which leads to the accumulation of cerebrospinal fluid in the ventricles (Allocco et al., 2019). Furthermore, knockout and mutation of the lgi1b gene cause severe hydrocephalus and developmental brain defects, including apoptosis, in zebrafish; however, the exact mechanism remains unclear (Teng et al., 2011). By knocking down or overexpressing DIPA (a family consisting of Ccdc85a, Ccdc85b, and Ccdc85c), the interaction of DIPA with p120 is attenuated, leading to subcortical heterotopia and hemorrhagic hydrocephalus. This result is similar to the mechanism in mice with hemorrhagic hydrocephalus caused by the Ccdc85c mutation (Markham et al., 2014). In addition, combined gene knockouts can lead to hydrocephalus in zebrafish, and combined knockouts of the calb2a and calb2b genes lead to severe hydrocephalus, which may be associated with cilia (Bhoyar et al., 2019). Hydrocephalus is also frequently observed in zebrafish models of other diseases, such as when the dcdc2 or slc41a1 genes, along with other NPHP genes, are knocked down to model renal cysts (Schueler et al., 2015; Hurd et al., 2013). Animals such as primates, pigs, dogs, and rabbits are predominantly used to develop models of acquired hydrocephalus; however, research in these species is often constrained by ethical considerations, individual differences, and other factors. Therefore, genetic interventions are less commonly studied in these animals. The genes associated with hydrocephalus identified in previous studies require further validation in these animals (particularly those with complex cortical structures) to provide more comprehensive insights. Although the zebrafish hydrocephalus model shares certain similarities with humans in terms of genes, brain structure, and cellular features, significant differences exist in their ventricular structures. Unlike mammals, the zebrafish brain ventricular system consists of only three cavities, lacking the Sylvian aqueduct that connects the third and fourth ventricles. Additionally, only two of these cavities meet the morphological criteria for ventricles. Therefore, there remains considerable debate regarding the efficacy of the zebrafish hydrocephalus model as a model for studying human hydrocephalus (Mogi et al., 2012; Wang et al., 2024). Moreover, there are differences in the molecular mechanisms underlying the development of the choroid plexus in humans and zebrafish. Given these considerations, further exploration of the zebrafish ventricle development process and molecular expression differences, as well as the development of highly specific zebrafish models, is crucial for advancing our understanding of the molecular mechanisms involved in the pathogenesis of hydrocephalus.

### 2.4 Genetic alterations in humans

The genes related to hydrocephalus in humans primarily involve neurodevelopment, cerebrospinal fluid circulation, and the structural and functional regulation of the ventricles and the blood-cerebrospinal fluid barrier. Although numerous genes have been found to be associated with the development of hydrocephalus in animal studies, only six genes have been definitively proven to be closely related to congenital hydrocephalus in humans: L1CAM, AP1S2, MPDZ, FOXJ1, SMARCC1, and CCDC88C. L1CAM encodes the L1 protein, a transmembrane glycoprotein belonging to the immunoglobulin superfamily. It is primarily expressed in neurons during development and plays a critical role in neuronal adhesion, axonal growth and guidance, and myelination. Studies have demonstrated that mutations in the L1CAM gene are a major cause of X-linked hydrocephalus, with these mutations located between the DXS52 and F8C loci. Affected patients often exhibit severe ventricular enlargement accompanied by profound intellectual disabilities and developmental delays (Liu et al., 2024; Ahmed et al., 2023). The AP1S2 gene encodes a subunit of the adaptor protein complex 1 (AP-1), which is critical for vesicle formation and trafficking within the Golgi apparatus. Clinically, mutations in AP1S2 are commonly associated with Fried-Pettigrew syndrome, characterized by hydrocephalus, intellectual disabilities, mild facial anomalies, and basal ganglia calcification (Saillour et al., 2007). Mutations in MPDZ and CCDC88C disrupt cerebrospinal fluid circulation and absorption by affecting cellular structure and function. MPDZ mutations impair intercellular junctions, while CCDC88C mutations compromise cytoskeletal stability and formation, collectively leading to hydrocephalus. Notably, these two genes share significant similarities in their neuropathological manifestations. Studies have shown that the protein encoded by MPDZ directly binds to the DAPLE protein encoded by CCDC88C, acting as a scaffold to promote ependymal cell planar polarity by inhibiting the non-canonical Wnt signaling pathway (Tessier et al., 2023). Heterozygous *de novo* mutations in the FOXJ1 gene encode a critical forkhead transcription factor essential for the formation of motile cilia (Hou et al., 2023; Wallmeier et al., 2019). These mutations result in ciliopathy, characterized by hydrocephalus and randomized left–right body asymmetry. The SMARCC1 gene encodes a chromatin remodeling protein, SWI/SNF-related matrix-associated actin-dependent chromatin regulator subfamily C member 1 (BAF155). Mutations in SMARCC1 are closely associated with CH phenotypes and neural tube development defects (Jin et al., 2020; Hourvitz et al., 2023). However, beyond these genes, there remain numerous others and their associated pathways that have been only minimally explored. To fully elucidate the molecular mechanisms underlying hydrocephalus, further research is imperative. Whether through the development of relevant animal models or large-scale clinical studies, deeper investigation holds the potential to uncover new genetic insights and therapeutic targets for congenital hydrocephalus. Although numerous genes associated with hydrocephalus have been identified through animal studies, research on the genetic basis of congenital hydrocephalus in humans remains in its early stages. While recent years have seen some breakthroughs, the scope and depth of related studies are still limited. Against this backdrop, large-scale clinical and translational genetic studies hold great promise not only for elucidating the molecular mechanisms underlying congenital hydrocephalus but also for providing more precise targets for prenatal genetic diagnosis in potential cases. These findings lay the foundation for early intervention and risk management in high-risk populations, thereby reducing the incidence of congenital hydrocephalus and significantly improving the prognosis and quality of life for affected children. Meanwhile these advancements could pave the way for the development of targeted interventions and precision medicine approaches, offering new treatment options to reduce the disease burden and improve the outcomes and quality of life for affected patients.

## 3 Molecular changes in hydrocephalus

Genes play a key role in congenital hydrocephalus, but hydrocephalus are also characterized by abnormal expression levels of proteins that are typically associated with this condition. The impact of altered expression of key proteins in the development of secondary and idiopathic hydrocephalus has been extensively examined, with studies showing that modulating or intervening in the expression of these proteins can significantly influence the progression of hydrocephalus. Among the proteins studied in the field of hydrocephalus are TGF-β, VEGF, Wnt, AQP, NF-κB, and NKCC.

### 3.1 The transforming growth factor-beta pathway

**Figure 1:**
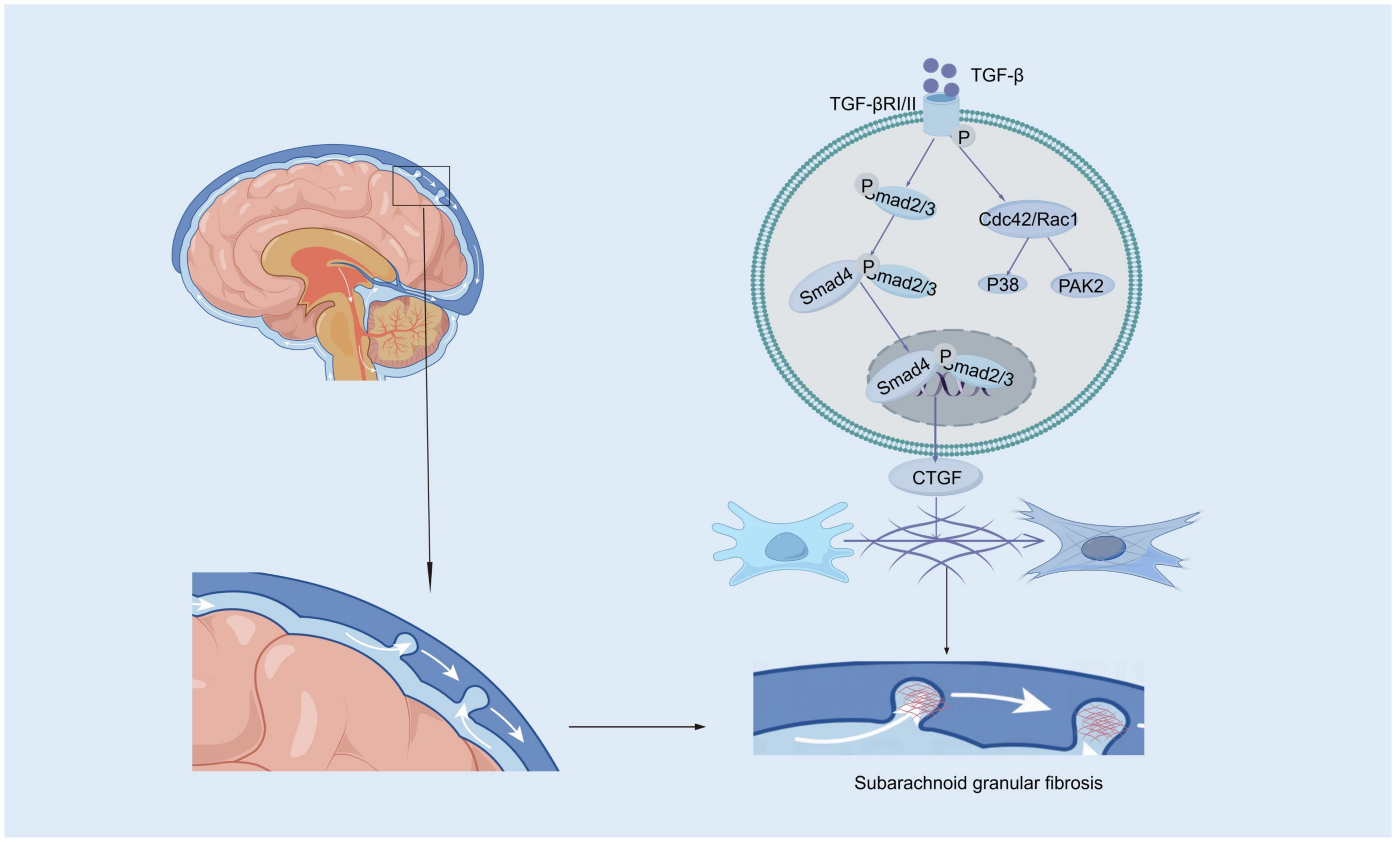
The canonical and non-canonical Smad signaling pathways induced by TGF-β. In the canonical Smad signaling pathway, TGF-β phosphorylates TGF-βRII, which recruits and phosphorylates TGF-βRI. The activated TGF-βRI subsequently phosphorylates Smad2 and Smad3 proteins. These activated Smad2 and Smad3 proteins then recruit Smad4 to form a complex, which translocates into the nucleus. Within the nucleus, the Smad complex interacts with specific DNA sequences and other transcription factors to promote the transcription and expression of target genes, such as CTGF (Connective Tissue Growth Factor). In the subarachnoid space, CTGF contributes to pia mater fibrosis by promoting the synthesis and deposition of extracellular matrix components. In the non-canonical Smad signaling pathway, the activated TGF-βRI/II complex can further activate Cdc42/Rac1, which in turn activates downstream factors such as the P38 and PAK2 signaling pathways.

Transforming growth factor-beta (TGF-β), a 25-kD nonglycosylated homodimer produced by various cell types, is a cytokine that is essential for the induction of the fibrotic response (Peng et al., 2022; Wang et al., 2018). There is strong evidence suggesting that intense contact occurs between TGF-β and hydrocephalus after stroke, especially in subarachnoid hemorrhage (Yang et al., 2022; Lee et al., 2013; Douglas et al., 2009). In the central nervous system (CNS), TGF-β is secreted by astrocytes, neurons, and microglia and has been reported to amplify fibrosis, leading to hydrocephalus in subarachnoid hemorrhage (SAH) (Lee et al., 2013; Robinson and Jantzie, 2022). The TGF-β family mediates signaling by binding to two serine/threonine kinase receptors on the cell surface, TGF-β RI and TGF-β RII. This interaction regulates extracellular matrix remodeling and drives the transition of fibroblasts into myofibroblasts, a critical process in fibrosis (Tzavlaki and Moustakas, 2020; Luo, 2017). In mouse models of SAH, TGF-β is expressed at high levels in CSF, leading to posttraumatic fibrotic scarring and angiogenesis, thereby resulting in chronic communicating hydrocephalus through the TGF-β/samds/CTGF signaling pathway (Figure 1). Dong et al. (2018) found that the expression of TGF-β1 in the CSF and brain parenchyma increased on the 21st day after SAH in a rat model. The protein levels of Smad2/3, pSmad2/3, and CTGF in the superficial tissues of the rat brain were significantly elevated following SAH, a response that was effectively suppressed by ICA II. Notably, TGF-β1 in CSF has been described to exhibit a biphasic response (Yan et al., 2016; Flood et al., 2001). The first peak of TGF-β1 primarily originates from an exogenous pathway, driven by the release of substantial amounts of pre-stored TGF-β1 from platelets during aSAH, which coincides with the process of platelet degranulation (Flood et al., 2001). The second peak is attributed to endogenous production mechanisms, wherein TGF-β1 acts both as a chemokine to attract inflammatory cells and platelets and synergistically interacts with other cytokines to stimulate local production of TGF-β1 in the CSF and choroid plexus (Yan et al., 2016; Kuo and Huang, 2021). Inhibiting the TGF-β1 signaling pathway could therefore mitigate chronic hydrocephalus in the aSAH model. LSKL peptide, a small molecule peptide and competitive antagonist of TGF-β1, suppresses TSP1-mediated TGF-β1 activity, thereby reducing subarachnoid fibrosis, preventing chronic hydrocephalus, and improving long-term neurocognitive outcomes after SAH (Liao et al., 2016). Similarly, Decorin, a natural antagonist of TGF-β, inhibits the downstream pathway by forming a complex with TGF-β and also acts as a competitive inhibitor (Derynck and Budi, 2019; Zhang et al., 2018). In a rat SAH model, Decorin effectively prevented extracellular matrix accumulation, subarachnoid fibrosis, and chronic hydrocephalus by inhibiting the heightened activity of the TGF-β1/Smad/CTGF axis (Yan et al., 2016). Moreover, Zhang et al. confirmed that HGF, MMP-9, and TGF-β1 may participate in the formation and prognosis of hydrocephalus after kaolin injection (Heep et al., 2004; Zhang et al., 2013). Cytologic research by Yue et al. (2016) reported that TGF-β1 activates the p38 signaling pathway in MMCs, which indicates that the p38 pathway is an important signaling pathway through which TGF-β1 induces the expression of CTGF. Studies have shown that the concentrations of TGF-β1 in the CSP of patients who suffer ICH-IVH or IVH-GMH are increased, especially in those with posthemorrhagic hydrocephalus (Whitelaw et al., 1999; Tsitouras and Sgouros, 2011). However, two studies reported that TGF-β inhibitors do not attenuate ventricular dilation after IVH in rats (Tubbs et al., 2003; Tuli et al., 2000). Taken together, the evidence discussed in this section suggests that inhibiting the TGF-β signaling pathway may be a powerful approach for treating hydrocephalus after hemorrhage.

### 3.2 Vascular endothelial growth factor

**Figure 2:**
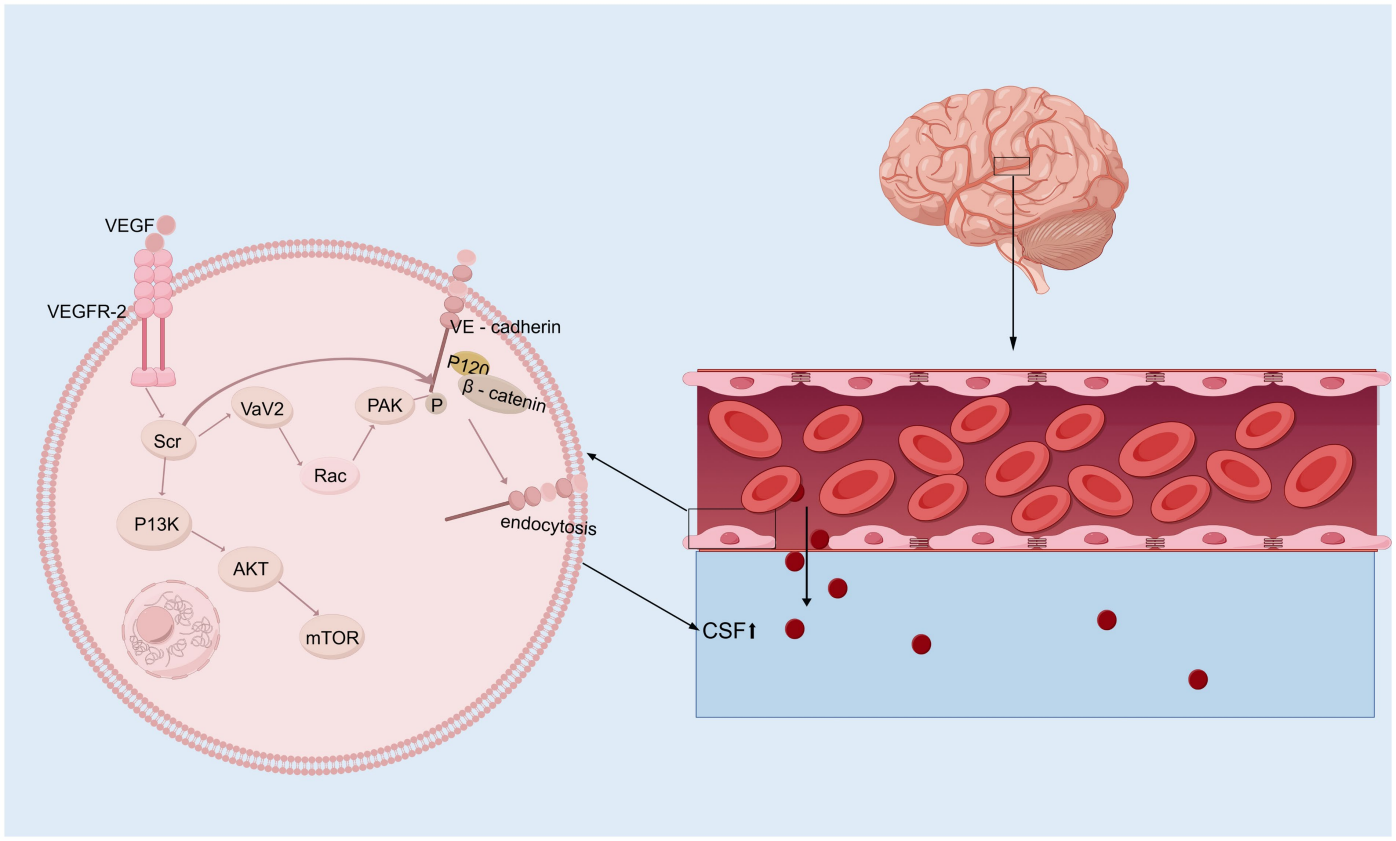
VEGF signaling pathway. Upon binding to its receptor VEGFR-2 on the surface of vascular endothelial cells, VEGF first activates Src kinase, which subsequently phosphorylates and activates Vav2. This activation facilitates the conversion of Rac from its GDP-bound inactive state to its GTP-bound active state. Activated Rac further activates PAK (p21-activated kinase). PAK, or alternatively the activated Src kinase, phosphorylates VE-cadherin (Vascular Endothelial Cadherin), allowing β-catenin to bind to its tail. This phosphorylation mediates VE-cadherin internalization, resulting in a “fenestration effect,” which contributes to the development of hydrocephalus. In addition, activated Src kinase can also mediate the PI3K/AKT/mTOR signaling pathway, initiating a series of downstream cascade reactions.

In the brain, VEGF is a potent growth factor that plays diverse roles in vasculogenesis and angiogenesis, mediates angiogenesis, neural migration, and neuroprotection, leading to angiogenesis and increased vascular permeability (Shim and Madsen, 2018; Grunewald et al., 2021). VEGF levels tend to be higher in the ventricular CSF of animals and patients with hydrocephalus (Yang et al., 2016; Naureen et al., 2014). Alternatively, VEGF/VEGFR-2 levels in the CP–CSF circulatory system may reflect the activity of the VEGF system in the brain, especially in periventricular areas (Yang et al., 2010). In rats, infusion of VEGF-A165 led to twofold enlargement of the ventricles, which had several other effects: elevation of VEGFR2 phosphorylation in the ependyma, alterations in β-catenin and E-cadherin levels, ependymal cell denudation, and altered ciliary staining on the ventricular surface (Shim et al., 2013). However, the ventricular response can vary in animal experiments depending on the infusion rate and how long the infusion rate is administered. Moreover, excess HB-EGF leads to a significant increase in VEGF and ventricular dilatation (Shim et al., 2016). VEGFR2 has been identified as the primary receptor for VEGF (Terman et al., 1992). The binding of VEGF induces VEGFR2 dimerization, which regulates the activation of Src kinase. This activation leads to the phosphorylation and internalization of VE-cadherin, while also reducing its interaction with associated proteins such as p120-catenin and β-catenin, thereby strengthening the endocytosis process (Dejana and Vestweber, 2013; Del Bigio, 2010; Delgado-Bellido et al., 2017; Li et al., 2016; Gavard and Gutkind, 2006; Xiao et al., 2005; Rahimi, 2017). Consequently, VE-cadherin expression on the cell membrane decreases, becomes unevenly distributed, and instead increases in the cytoplasm. This redistribution disrupts intercellular junctions, exacerbates the open-window effect, compromises the blood–brain barrier (BBB), and ultimately contributes to hydrocephalus development (Shen et al., 2022). Additionally, VEGFR2 dimerization enhances the activation of the small GTPase Rac through Src-dependent phosphorylation of the guanine nucleotide exchange factor Vav2. Activated Rac promotes the p21-activated kinase (PAK)-mediated phosphorylation of conserved motifs in the intracellular tail of VE-cadherin. This phosphorylation recruits β-arrestin-2 to the serine-phosphorylated VE-cadherin, further promoting its internalization into clathrin-coated vesicles. The resulting disassembly of intercellular junctions further disrupts the BBB and contributes to hydrocephalus development (Figure 2) (Apte et al., 2019; Lolansen et al., 2021). Interestingly, metformin can inhibit VEGF/VEGFR2/p-Src pathway activation, reverse the internalization of VE-cadherin, and ameliorate IVH-induced hydrocephalus in a rat model (Shen et al., 2022). Although VEGF is a potential therapeutic target for hydrocephalus, its role has been explored in relatively few studies, and its specific mechanisms have yet to be fully elucidated. Moreover, the efficacy of targeted therapies against VEGF still requires further investigation through translational research.

### 3.3 Wnt signaling pathway

**Figure 3:**
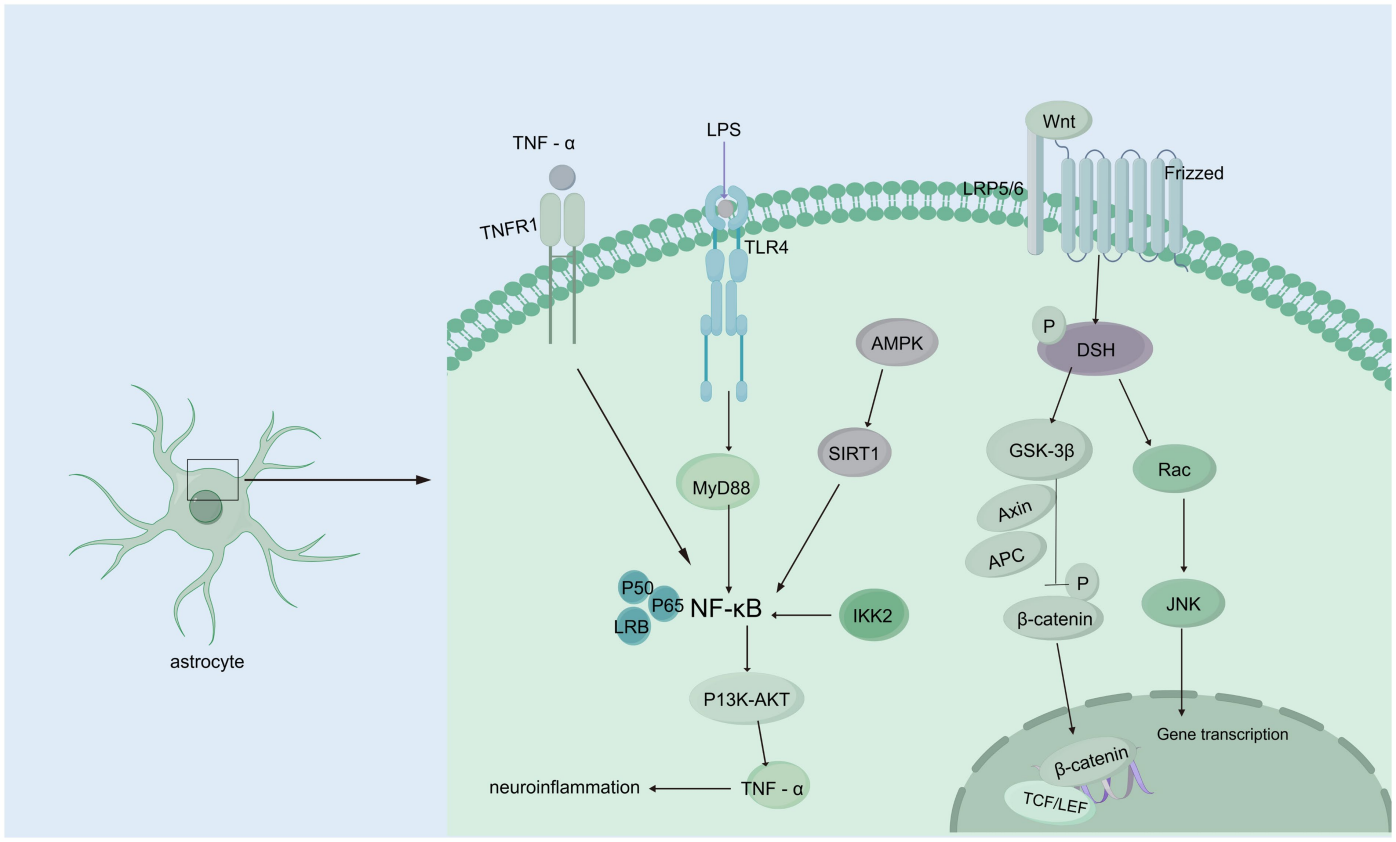
Wnt/β-catenin and NF-κB signaling pathways. Wnt/β-catenin signaling pathway: When Wnt ligands bind to the Frizzled receptor on the membrane of astrocytes, they activate DSH (Disheveled) protein, initiating downstream signaling. DSH inhibits GSK-3β (glycogen synthase kinase-3β) activity, preventing β-catenin phosphorylation. This leads to the accumulation of β-catenin in the cytoplasm, allowing it to translocate to the nucleus, where it binds to transcription factors such as TCF/LEF, regulating gene expression and interfering with CSF circulation and absorption, which contributes to hydrocephalus. Additionally, activated DSH can activate Rac, which further activates the JNK signaling pathway, affecting CSF homeostasis. NF-κB signaling pathway: TNF-*α* binds to the TNFR1 receptor, directly activating NF-κB signaling; TLR4 recognizes endogenous or exogenous ligands, activating NF-κB through the MyD88-dependent pathway. AMPK activation, caused by cellular energy imbalance, stimulates SIRT1, which subsequently activates NF-κB signaling. IKK2 activation can also trigger NF-κB signaling. Activated NF-κB regulates downstream PI3K/AKT signaling, upregulating TNF-α, forming a positive feedback loop that exacerbates inflammation and further contributes to hydrocephalus development.

The Wnt/β-catenin signaling pathway initiates a signaling cascade that is critical for the normal development of multiple organ systems; furthermore, this pathway plays a crucial role throughout all stages of brain development, such as neurostem cell development and subventricular zone development, and it is linked to many neurological disorders (Song et al., 2021; Clevers and Nusse, 2012). In the presence of the Wnt/β-catenin signaling pathway, the binding of Wnts to a frizzled receptor and low-density lipoprotein receptor-related protein 5/6 (LRP5/6) coreceptor triggers the recruitment of the cytoplasmic component, which is dishevelled and thus inhibits the phosphorylation of β-catenin via glycogen synthase kinase three beta (GSK-3b) (Noelanders and Vleminckx, 2017; Nusse and Clevers, 2017; Zhao et al., 2022). In the hydrocephalus rat model induced by low-concentration kaolin (3%), the Wnt signaling pathway was activated, leading to reactive gliosis, which can be reversed by secreted frizzled-related protein 1 (sFRP-l). In contrast, the development of hydrocephalus is delayed (Xu et al., 2015; Suryaningtyas et al., 2020). Moreover, the increase in Wnt/Wnt3a mRNA and protein expression was significant in rats with hydrocephalus induced by intraventricular injection of autologous blood, and deferoxamine alleviated this increase, suggesting that iron is a vital factor that activates the Wnt signaling pathway (Meng et al., 2015). A study on the role of the R595H-Trim71 mutation associated with congenital hydrocephalus in neural differentiation has shown that regulating the Wnt/β-catenin signaling pathway can effectively improve the neural differentiation defects in R595H-Trim71 mutant cells. These findings suggest that the Trim71 mutation may play a key role in the pathogenesis of congenital hydrocephalus through specific pathological mechanisms, providing a new direction for the development of precise therapeutic strategies for congenital hydrocephalus (Figure 3) (Liu et al., 2023; Cuevas et al., 2015).Further research is needed to establish definitive evidence of the relationships among ferroptosis, Wnt signaling, and hydrocephalus. Interestingly, the Wnt signaling pathway plays a pivotal role in organ fibrosis, such as renal fibrosis and liver fibrosis, and the mechanism of the Wnt signaling pathway in flexural meningeal fibrosis in hydrocephalus remains unclear.

### 3.4 Aquaporins

Aquaporins (AQPs) are integral membrane proteins that facilitate selective water and solute transport across cell membranes and maintain cellular homeostasis and fluid balance in neural compartments (Filippidis et al., 2016; Toader et al., 2023). The expression profiles of AQPs, particularly AQP4 and AQP1, have been demonstrated to be significantly expressed in the CNS, and dysregulated AQP expression is implicated in various brain pathologies (Filippidis et al., 2016). In the CNS, AQP4 is expressed predominantly in subpial astrocyte processes, which form the glial-limiting membrane, perivascular astrocyte endfeet, and the basolateral membrane of the ependymal and subependymal regions (Kitchen et al., 2020). AQP4 is variable in the early stage but higher in the later stage, indicating that it compensates for reducing the production of CSF and the mechanism for the clearance of excess interstitial fluid in hydrocephalus (Mao et al., 2006; Gonzalez-Marrero et al., 2022). In the spontaneously hypertensive rat model, AQP4 expression was significantly lower in ventricular cells and subventricular astrocytes of 12-month-old spontaneously hypertensive rats compared to control rats and 6-month-old spontaneously hypertensive rats. This suggests that changes in AQP4 expression in spontaneously hypertensive rats may play a more significant role in obstructing the pathway of CSF from the ventricles to the parenchyma, rather than merely decreasing the volume of ventricular CSF to prevent edema (Paul et al., 2011). In congenitally hydrocephalic H-Tx rats, a significant increase in the cerebral cortical expression of AQP4 was observed (Shen et al., 2006). If the gene is knocked out in mice, it disrupts gap junctions, which alters the ventricular zone and cerebrospinal fluid flow, leading to hydrocephalus development (Li et al., 2009). Significantly increased expression of AQP4 has been reported in several models, including the kaolin-induced hydrocephalus model in rats, Texas rats with congenital hydrocephalus, dogs with idiopathic obstructive hydrocephalus, and a rat model of inflammatory obstructive hydrocephalus. This increase in AQP4 expression was strongly correlated with the severity of hydrocephalus (Paul et al., 2011; Schmidt et al., 2016; Tourdias et al., 2009). Interestingly, the expression of AQP4 was not detected, which can be explained by the experimental results of Aghayev et al., who reported that the expression of AQP4 in mild hydrocephalus is not elevated (Aghayev et al., 2012). In recent years, the role of AQP4 in the glymphatic system has been gradually elucidated. Alterations in the glymphatic system associated with the downregulation or redistribution of AQP4 appear to play a role in the etiology of idiopathic normal-pressure hydrocephalus, but it remains unclear whether there is are direct associations between the glymphatic system, AQP4, and hydrocephalus. AQP1 is predominantly localized to the ventricular-facing plasma membrane of choroid plexus epithelial cells. Experimental evidence has shown that knockdown of AQP1 reduces osmotically-driven water permeability in choroid plexus epithelial cells, leading to a decrease in CSF production and a reduction in intraventricular pressure. These findings suggest that AQP1 plays a critical role in the regulation of CSF production (Zanotto et al., 2017). In the rat model of kaolin-induced hydrocephalus, AQP 1 expression decreased dramatically in the early stages of hydrocephalus by about 50% through the mechanism of endocytosis restoration (Wang et al., 2011) Additionally, a marked reduction in AQP1 expression in the choroid plexus epithelium was observed in spontaneously hypertensive rats. In Texas rats with congenital hydrocephalus, choroidal AQP1 expression was reduced early in life but normalized by postnatal day 26, prior to death (Paul et al., 2011). However, experiments involving AQP1 knockout and AQP4 knockout mice, which were injected intravenously with O17-labeled water (H2O17), revealed that water entering the lateral ventricles was significantly reduced in AQP4 knockout mice but not in AQP1 knockout mice, suggesting that AQP4 plays a critical role in cerebrospinal fluid formation (Igarashi et al., 2014). Moreover, the upregulation of AQP5 and downregulation of AQP1 with an apical localization in choroid plexus epithelial cells were observed in hydrocephalus following IVH (Sveinsdottir et al., 2014). The main functions of AQP5 include regulating water permeability, paracellular water transport, and cytoskeletal organization and stability (Express Group, 2010; Sidhaye et al., 2008; Sidhaye et al., 2012). In a rabbit model of IVH with posthemorrhagic ventricular dilatation and *in vitro* cultured human choroid plexus epithelial cells treated with posthemorrhagic cerebrospinal fluid and hemoglobin chloride, it was observed that AQP1 mRNA, a key regulator of CSF production, was downregulated. However, the protein level of apical epithelial cell-localized AQP1 was upregulated. Additionally, AQP5 was expressed in the choroid plexus, with both its mRNA expression and protein levels increasing during posthemorrhagic ventricular dilatation, specifically in apical epithelial cell localization (Sveinsdottir et al., 2014). AQP9 was expressed in astrocytes, cerebellar neurons, limbic vascular endothelium, glial border membrane, hypothalamic monolayer cells, and CA2 in the hippocampus (Badaut and Regli, 2004; Badaut et al., 2002). AQP11 was localized to the choroid plexus epithelium and cerebral capillary endothelium, suggesting its potential involvement in water transport within the choroid plexus and across the BBB in the brain (Filippidis et al., 2016). In addition, the relationships among AQP4, AQP1, and CSF transport have been progressively elucidated, revealing their critical roles in maintaining CSF homeostasis. However, despite the growing understanding of the functions of AQP5, AQP9, and AQP11 in CSF transport, their specific mechanisms in the development and progression of hydrocephalus still require further investigation, with the aim of providing new targets for the early diagnosis and treatment of hydrocephalus.

### 3.5 Nuclear factor-κB

Many neurological diseases, including hydrocephalus, are associated with neuroinflammation, and a significant regulator of inflammation is nuclear factor-κB (NF-κB). The expression of constitutively active IKK2 in astrocytes induces NF-κB activation, causing hippocampal malformation and resulting in early postnatal hydrocephalus associated with a lack of ependymal cilia (Lattke et al., 2012). Interestingly, NF-κB activation in astrocytes causes hydrocephalus only in the developing brain. NF-κB signaling in ependymal cells of the ventricle is increased following IVH (Simard et al., 2011). Moreover, in a kaolin-induced hydrocephalus model, toll-like receptor 4 (TLR4)-NF-κB signaling in the choroid plexus epithelium (CPE) stimulates CSF hypersecretion through the SPAK-NKCC1 cotransporter complex, thereby uncovering a novel kinase-regulated mechanism of CSF secretion (Heep et al., 2004; Xu et al., 2024) and the expression level of the TLR4–NF-κB signaling pathway was increased significantly in hydrocephalus after IVH. In an IVH rat model, TAK-242, which is a TLR4 inhibitor, effectively downregulated the TLR4–NF-κB signaling pathway, fibronectin, and laminin and significantly alleviated ventriculomegaly after IVH (Lin et al., 2022). In post-IVH hydrocephalus, the phospho-NF-κB (p-NF-κB) signaling pathway is activated, and metformin attenuates neuroinflammation and subsequent fibrosis after IVH by regulating the AMPK/SIRT1/NF-κB pathway (Cao et al., 2023).Additionally, it has been demonstrated that the aggregation of choroid plexus (ChP) macrophages exacerbates the inflammatory response of ChP epithelial cells via the TNF-*α*/TNFR1/NF-κB signaling cascade, leading to an increased secretion of CSF (Wang et al., 2024) In experimental models using ChP epithelial (CPE) cells to simulate the inflammatory conditions of IVH, it was observed that NKCC1, a key transporter involved in CSF secretion from the choroid plexus, is predominantly activated by interleukin-6 (IL-6) (Figure 3) (Johnsen et al., 2023).In addition, some studies have shown that NF-κB causes neuroinflammation via the PI3K-AKT/TNFAIP3 pathway in an experimental germinal matrix hemorrhage rat model, which can be reversed by rh-IFN-α (Li et al., 2020). However, it remains unclear whether inhibiting NF-κB through the JAK1-STAT1/TRAF3 pathway attenuates post-IVH hydrocephalus. Compelling evidence has confirmed that signaling pathways involving NF-κB are crucial for the pathogenesis of posthemorrhagic hydrocephalus (PPH) and may be effective targets for treating PPH. Research on the role of NF-κB in PPH is still limited, and its specific mechanisms have not been fully elucidated. Therefore, there is an urgent need for more systematic and in-depth basic and translational research to reveal the critical role of NF-κB in the development and progression of PPH, providing a theoretical basis and innovative ideas for the development of targeted therapeutic drugs for PPH.

### 3.6 Na^+^/K^+^/2Cl cotransporter

**Figure 4:**
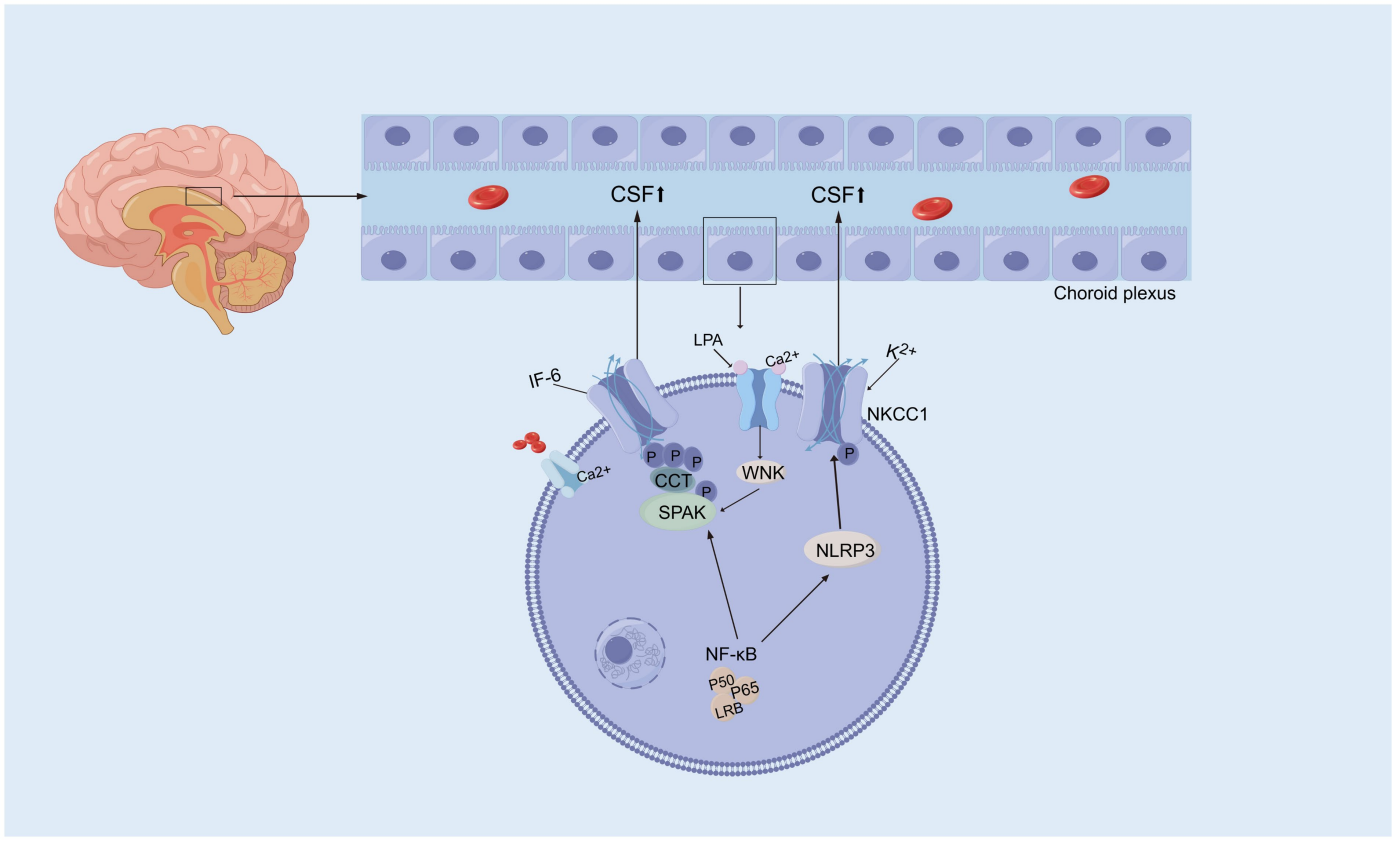
Activation of Na^+^/K^+^/2Cl cotransporter. Activated NF-κB initiates a signaling cascade that eventually activates SPAK, which phosphorylates and activates NKCC1, resulting in excessive CSF secretion and contributing to hydrocephalus. Moreover, activated NF-κB also influences NKCC1 activity by modulating the NLRP3 inflammasome, leading to abnormal CSF secretion and the promotion of hydrocephalus. Serum lipid LPA directly acts on TRPV4, mediating Ca^2+^ influx, which regulates the WNK-SPAK-mediated phosphorylation of NKCC1. This results in an increased CSF secretion rate and ventricular enlargement.

Na^+^/K^+^/2Cl^−^ cotransporters (NKCCs) are located on the apical membrane of the choroid plexus in the central nervous system as essential mechanisms of cell volume regulation and contribute to approximately half of the production of CSF (Loscher and Kaila, 2022; Karimy et al., 2017). NKCC1 contributes to CSF formation by transporting Na^+^, K^+^, and Cl^−^ transmembrane coupled water, which enables water to be transported (Lolansen et al., 2022). Bumetanide, a chloride importer antagonist of NKCC1, has been shown to attenuate hydrocephalus after IVH (Simard et al., 2010). And attenuated abnormal CPE secretion and hydrocephalus by inhibiting TLR4 / NF-κB / NKCC1 and AQP1 (Xu et al., 2024; Johnsen et al., 2023). An experimental study of genetic risk for hydrocephalus revealed that loss of the Ptpn20 gene in H-TX rats resulted in the development of communicating hydrocephalus, and the same result was observed in Ptpn20−/− mice, in which NKCC1 phosphorylation is maintained in choroid plexus epithelial cells (Xu et al., 2022). It has been demonstrated that serum lipid LPA enters the ventricular system during hemorrhagic events and acts directly on TRPV4, and activation of TRPV4 leads to hyperactivation of NKCC1, such that the elevated rate of CSF secretion appears to contribute to the ensuing ventricular dilation leading to PHH (Toft-Bertelsen et al., 2022), suggesting that the development of PHH is associated with CSF hypersecretion in part because of choroidal plexus Na+/K+ − ATPase and NKCC1 hyperactivation, but the underlying molecular coupling remains to be explored (Lolansen et al., 2022). In a hydrocephalus rat model after IVH, the phosphorylation of NKCC1 was increased via the activation of NLRP3 inflammasome components, which indicated the involvement of the NLRP3/p-NKCC1 pathway and Na^+^ and K^+^ flux in the PPH (Zhang et al., 2022). Intraventricular blood increases CSF [K^+^] and triggers cytosolic calcium activity in epithelial cells, followed by NKCC1 activation (Johnsen et al., 2023). Interestingly, with elevated CSF [K^+^], NKCC1 activation leads to a net flux of ions and osmotically obliges water movement from the CSF into the choroid plexus, resulting in compensated PHHs. Choroid plexus-targeted NKCC1 overexpression can be used to treat acute hydrocephalus after IVH (Sadegh et al., 2023). Moreover, overexpression of NKCC1 in the choroid plexus results in increased CSF [K+] clearance, which reduces ventriculomegaly in the critical period during postnatal neurodevelopment in mice (Xu et al., 2021). The evidence above suggests that NKCC1 plays a vital role in inflammation-dependent cerebrospinal fluid hypersecretion by the choroid plexus epithelium in posthemorrhagic hydrocephalus (Figure 4). Interestingly, CSF containing elevated levels of a subset of inflammatory markers expressed in the choroid plexus of rats and humans did not activate NKCC1 in iNPH patients (Lolansen et al., 2021). Although the overexpression of NKCC1 in posthemorrhagic hydrocephalus has been observed, its specific mechanism remains unclear. Therefore, it is crucial to further investigate how NKCC1 overexpression affects CSF secretion and the development of hydrocephalus, particularly by exploring its relationship with CSF dynamics at the molecular level. This will not only deepen our understanding of its role in the pathological process but also provide a theoretical foundation for developing targeted therapeutic strategies. Additionally, future research should focus on developing NKCC1-targeted interventions, offering new therapeutic targets for the clinical treatment of posthemorrhagic hydrocephalus and improving patient outcomes.

## 4 Conclusion and future perspectives

In summary, the development of hydrocephalus is a complex pathological process involving various genetic and molecular alterations. Any congenital or acquired factors that impact the structure and function of the ventricular system or disrupt the production, circulation, and absorption of cerebrospinal fluid can independently or synergistically lead to the onset of hydrocephalus. Congenital hydrocephalus is predominantly associated with abnormalities in the development of the ventricular system, whereas secondary hydrocephalus is linked to the dysregulation of multiple molecular pathways, including those related to inflammation, fibrosis, and injury.

Recent advancements in artificial intelligence (AI) and machine learning (ML) are playing an increasingly important role in understanding and predicting hydrocephalus. AI and ML models have demonstrated promising potential in analyzing imaging data to detect early changes in the brain associated with hydrocephalus, helping clinicians make more accurate diagnoses. Moreover, AI-driven predictive models are being developed to assess the functionality of ventriculoperitoneal shunts and provide real-time monitoring of disease progression. This technological shift not only enhances the detection of hydrocephalus but may also offers new possibilities for early intervention, especially in high-risk populations, by identifying critical molecular biomarkers and pathways associated with the disease. Integrating these approaches with molecular studies could eventually lead to the identification of novel targets for pharmacological treatments, optimizing the management of both congenital and secondary hydrocephalus.

Currently, several preclinical studies targeting the molecular mechanisms underlying secondary hydrocephalus have demonstrated promising results in preventing hydrocephalus and mitigating related damage. These findings suggest that the development of detection and intervention strategies targeting specific proteins or pathways, potentially assisted by AI and ML techniques, may hold significant potential for identifying high-risk patients and guiding the pharmacological treatment of secondary hydrocephalus. Furthermore, targeting gene alterations associated with hydrocephalus may play a critical role in screening for congenital hydrocephalus, thus offering new opportunities for early intervention.

To date, no drug has emerged from clinical trials that significantly improves hydrocephalus symptoms or reverses the progression of the disease. The complex pathological mechanisms of hydrocephalus make it challenging for a single drug to comprehensively regulate these mechanisms, resulting in limited therapeutic effectiveness. Furthermore, many drugs target widely distributed signaling pathways, which can lead to unpredictable side effects, thereby restricting the clinical application of these drugs. The integration of advanced methodologies could potentially guide the development of more specific treatments, enhancing therapeutic outcomes and reducing side effects. Further research into molecular mechanisms, improved diagnostics, and the development of targeted drugs is needed to fill this gap, ultimately improving the management and prognosis of hydrocephalus.
